# Application of an Intelligent Sensor and Active Packaging System Based on the Bacterial Cellulose of *Acetobacter xylinum* to Meat Products

**DOI:** 10.3390/s22020544

**Published:** 2022-01-11

**Authors:** Andi Dirpan, Muspirah Djalal, Irma Kamaruddin

**Affiliations:** Department of Agricultural Technology, Hasanuddin University, Makassar 90245, Indonesia; muspirah_djalal@agri.unhas.ac.id (M.D.); irmakama9@gmail.com (I.K.)

**Keywords:** smart sensor, smart packaging, meat shelf-life, food quality

## Abstract

Combining intelligent and active packaging serves the dual purpose of detecting color changes in food that reflect changes in its quality and prolonging its shelf life. This study developed an intelligent and active packaging system made from the cellulose of *Acetobacter xylinum* and assessed its ability to detect changes in the quality and to increase shelf-life of packaged fresh beef. The properties of the intelligent packaging’s sensor and active packaging films were determined. The application of this system to fresh beef stored at room temperature (28 ± 2 °C) for 24 h was tested. The color of the bromothymol blue (BTB) solution (pH 2.75) in the indicator of the intelligent packaging system changed from orange to dark green to indicate that beef quality changed from fresh to rotten. The meat treated with the active packaging with 10% and 15% garlic extract decayed on the 16th h. In contrast, the meat treated with the active packaging without the garlic extracts rotted on the 12th h. The shift in the indicator’s color was linearly related to the total plate count (TPC), total volatile basic nitrogen (TVBN), and pH of the meat packaged using the active packaging system. Therefore, BTB solution (pH 2.75) can be used as an intelligent packaging indicator that will allow consumers to assess the quality of packaged meat easily. As an antimicrobial agent, the addition of 10–15% garlic extract to the active packaging films can help delay the spoilage of packaged beef.

## 1. Introduction

Global beef consumption is predicted to rise as the world population and family income increase, particularly in developing Asian countries [1,2,3]. By 2030, worldwide meat consumption and availability are expected to increase by 14% and 5.9%, respectively, over the average of the 2018–2020 period [3]. Thus, the expected increase in meat consumption must be complemented by improvements in the quality of fresh meat produced. One aspect affecting the quality and characteristics of meat is the material and packaging technologies used [4]. Meat is a perishable item that rapidly spoils when stored above the optimum temperature range (below −17 to 4 °C) [5,6]. However, in traditional markets, meat is displayed at room temperature without packaging, a practice that might accelerate microbial contamination and cause rapid quality degradation. Even in supermarkets where meat is maintained in cold temperatures, standard meat packaging still prevents consumers from subjectively determining the quality of meat. Thus, meat packaging must have additional functions that will prevent quality degradation due to microbial contamination and will help consumers to determine the quality of packaged meat easily [7]. Conventional meat packaging can be designed to perform dual functions through intelligent and active packaging systems. 

Intelligent packaging is a term that refers to sensors in the form of indicators that monitor and provide information on the quality of the food contained within the packaging via color changes caused by chemical reactions between the indicators and the products of microbial metabolism or changes in the chemical composition of the food [8,9]. During storage, the chemical components of meat degrade into volatile compounds because of microbial activity, thereby increasing the value of total volatile base nitrogen (TVBN) [10,11]. Accumulation of TVBN increases the pH of the packaging system, which is detected by the indicator, resulting in a visible color shift in the indicator [11,12]. Intelligent packaging allows easier monitoring of packed products during transportation and storage [7]. Moreover, it provides a more accurate estimate of product condition than conventional expiration labels [12]. Color-based pH indicator solutions are widely used as intelligent indicators. Dirpan et al. [13] developed bromophenol blue as an intelligent indicator dye for mangoes. Hidayat et al. [14] used two types of color indicators with predetermined concentrations, namely, phenol red and bromothymol blue, to assess the freshness of meat packaging. Intelligent packaging indicators based on natural pigments are being developed, such as intelligent packaging films that include anthocyanin-loaded Lycium ruthenicum nanocomplexes in starch/polyvinyl alcohol mixtures (PVA) [15], as well as anthocyanins from saffron petals immobilized in chitosan nanofibers and methyl cellulose matrix [16].

Active packaging refers to the integration of particular additives into a packaging system for the purpose of extending the shelf life, preserving the quality, and ensuring the safety of food products. Antimicrobial agents are used as components of active packaging additives to extend product shelf life. The volatile bioactive compounds in active packaging evaporate or diffuse onto the food surface, where they limit the growth of microbes and thus delay spoilage [17,18]. This strategy is more effective than coating bioactive compounds onto the food surface [19]. The safest, cheapest, and most readily available antimicrobial agents for use in active packaging are essential oils. Pranoto et al. [20] produced antimicrobial alginate edible films by incorporating the essential oils of garlic. They reported that these films substantially inhibited the growth of *Staphylococcus aureus* and *Bacillus cereus* in meat. Vishnu et al. [21] utilized the essential oils of *Plectranthus amboinicus* in a chitosan-based active packaging to restrict antimicrobial activity. 

Intelligent and active packaging can be merged into a single packaging system. Julyaningsih et al. [22] combined an intelligent packaging system based on methyl red and bromothymol blue (BTB) indicator with an active packaging system based on lemongrass oil as a component of tuna fish fillet packaging. Yao et al. [23] developed an active and intelligent packaging system based on starch, PVA, and betacyanins from various types of plants for shrimp packaging. In general, an active packaging that contains antimicrobial agents and an intelligent packaging that contains indicator solutions are immobilized in a polymer. Compared with synthetic polymers or plant cellulose, the bacterial cellulose fermented by *Acetobacter xylinum* has a unique nanofibrillar structure and superior physical properties, suggesting that it has the potential to serve as a basis for developing an intelligent and active packaging system [24,25]. Bacterial cellulose has received interest as a component of active packaging owing to its biodegradability, high water-holding capacity so that it can be employed entirely as a polymer for immobilizing color solutions in intelligent packaging indicators [26]. Moreover, bacterial cellulose possess great potential as an antimicrobial agent carrier in order for it to be utilized as an ingredient in the production of active packaging films [27].

The development of packaging systems with additional functions is advancing. To promote this innovation, this study aimed to maximize the potential of intelligent and active packaging by combining them into a single packaging system based on a bacterial cellulose membrane biopolymer to enhance the quality of packaged meat and help consumers to determine meat freshness easily.

## 2. Materials and Methods

### 2.1. Materials

The main ingredients used in the intelligent and active packaging system developed herein were the bacterial cellulose produced by *A. xylinum*, which was fermented in natural media of coconut water. Beef tenderloin was purchased from a slaughterhouse in Tamangapa Raya. Coconut water and garlic (*Allium sativum*) were purchased from a local market. Food-grade ammonium sulfate (Lianyungang Zhonghong Chemical Co., Ltd., Lianyungang, China, CAS No: 7783-20-2), yeast extract (Merck, Darmstadt, Germany, CAS No: 8013-01-2), 96% acetic acid (Brenntag Inc, Essen, Germany, CAS No: 64-19-7), *A. xylinum* culture, 5% 1 N NaOH (Brenntag Inc, CAS No: 1310-73-2), sucrose, Bromothymol Blue (BTB) (Merck, Darmstadt, Germany, CAS No: 76-59-5), alcohol (Sd Fine Chem Limited, Chennai, Tamil Nadu, India), aquabides, aquades (Rofa Laboratorium Centre, Bandung, Indonesia) Tashiro’s indicator (0.1% Methyl Red [Merck, Darmstadt, Germany, Cas No: 493-52-7] and 0.1% BTB at a ratio of 2:1), 7% trichloroacetic acid (TCA) (Merck, Darmstadt, Germany), Nutrient Agar (NA) (Merck, Darmstadt, Germany), glycerol (Merck, Darmstadt, Germany, CAS No: 56-81-5), food-grade carboxymethyl–cellulose (CMC) (Foodchem, Shanghai, China, E466), and corn starch were used.

### 2.2. Methods

#### 2.2.1. Production of Bacterial Cellulose from *A. xylinum*

Based on our previous research Dirpan et al. [7], 5% (*w/v*) of food grade Ammonium Sulfate is the best source of Nitrogen in *Acetobacter xylinum* growth media to produce optimal bacterial cellulose membranes. Determination of the composition and type of Nitrogen source. Then, purification of bacterial cellulose was done by removal from the fermentation medium, rinsed in running water, and then soaked for 2 days with periodic water changes. The cellulose was also soaked in 70% alcohol for 1 min, heated to 100 °C in distilled water for 20 min, and reheated in 1 N 5% NaOH solution at 100 °C for 60 min to remove the remaining bacterial cells and substrate attached to the cellulose layer. Afterward, the cellulose was rinsed with running water and soaked in periodically changed water for 24 h until pH reached 7. The purified cellulose appeared transparent [7].

#### 2.2.2. Production of Intelligent Packaging 

First, preparation of the indicator solution. BTB indicator solution was chosen for this study because a previous work established this solution as the indicator with the most visually identifiable color change reaction [7]. First, 1% BTB solution (*b/v*) was prepared in 95% ethanol. Then, the pH of the BTB solution was decreased to 2.74 by adding 20% acetic acid. Finally, the BTB solution was stored in a closed container. Second, production of intelligent packaging indicator label. The purified cellulose film was kept in a filter cloth for 24 h to decrease its water content. Half-dried cellulose was cut into 1.5 cm × 4 cm strips and pushed flat against the surface of a Pyrex glass. The cellulose was dried for 30 min at 70 °C until the moisture content reaches 6%. A total of 35 mL of I BTB indicator solution was then absorbed into a dry cellulose via centrifugation at 3000 rpm for 15 min. When the color indicator was successfully absorbed, the BTB indicator solution imparted an orange hue to the cellulose. Afterward, the cellulose was rinsed with distilled water to eliminate any unbound color indicators and then dried [26,28].

#### 2.2.3. Production of Active Packaging Film

First, the production of garlic extract as an active element. The method applied in this research referred to Yolanda et al. [29] with a slight modification. A total of 500 g of garlic was peeled, washed under running water until clean, drained, and then mashed. The minced garlic was extracted via the maceration method by immersing the finely ground garlic in 96% alcohol at a ratio of 1:4 (garlic: alcohol) for 4 days at 3–5 °C and periodically homogenized using a water bath shaker. Afterward, the extract was filtered using a filter paper and then concentrated using a rotary evaporator at 50 rpm at 40 °C to obtain a thick extract. Second, production of active packaging film. The method used referred to Iriani et al. and Indrarti et al. [19,30] with a slight modification. The bacterial cellulose was crushed to form a cellulose pulp. A cellulose suspension was prepared using 30% chitosan (*w*/*w*), 10% CMC (*w*/*w*), and 15% corn starch (*w*/*w*) of cellulose dry weight. The suspension was heated at 50 °C for 60 min with a hot plate stirrer until thoroughly suspended. At the 50th min, 30% glycerol (*w*/*w*) was added. Additionally, the garlic extract was added at quantities of 0% (as the control), 5%, 10%, and 15% (*v*/*v*) immediately after the final heating step. Subsequently, 60 g of the suspension was then placed onto a glass plate and dried for 48 h at 37 °C. Finally, the suspension was cooled to room temperature, removed from the glass plate, wrapped in aluminum foil, and placed in a desiccator.

#### 2.2.4. Application of the Intelligent and Active Packaging Indicators to Fresh Beef

**Figure 1:**
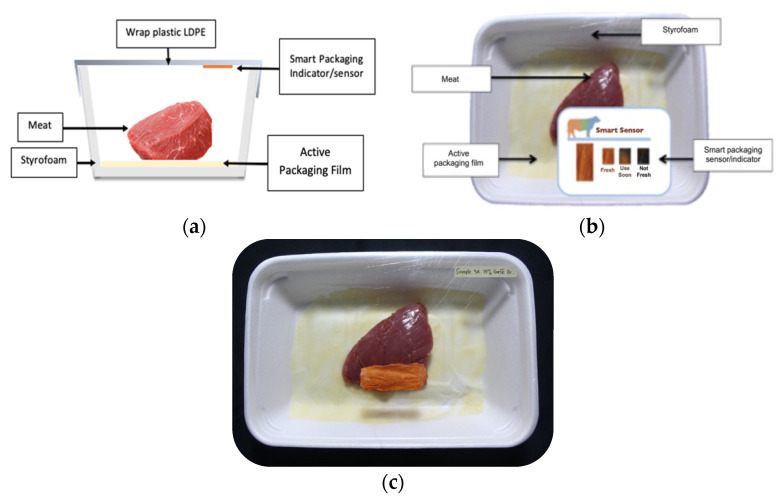
(**a**) Design of the intelligent and active packaging system; (**b**) prototype of the intelligent packaging; (**c**) and its application on fresh beef.

Fresh beef tenderloin was collected from a slaughterhouse in Tamangapa Raya Makassar 1 h after the cow was slaughtered. It was immediately placed in a special food box and put into a 38 cm × 29 cm × 30 cm Styrofoam box filled with ice crystals. The samples were promptly transported to the laboratory and processed into 200 g/pack pieces. The meat was packaged in a Styrofoam tray (1.05 g/cm^3^) coated with the active packaging film on a Styrofoam base, and an intelligent packaging indicator label was attached to the LDPE plastic wrap film that covered the Styrofoam container (Figure 1). The samples were maintained at room temperature (28 ± 2 °C) with normal light exposure for 24 h.

During the entire storage period, the intelligent packaging label changed its color three times that corresponded to three phases of the meat samples’ level of quality (Figure 1b). In phase I, its color was orange, indicating that the meat samples were still fresh. In phase II, its color was green with an orange hue, suggesting that the meat samples should be consumed immediately (Use soon). In phase III, its color was dark green, denoting that the meat samples were already spoiled (Not Fresh).

#### 2.2.5. Observation Parameters

##### Measurement of Intelligent Packaging Indicator Color Response on Meat

The color of the intelligent packaging indicators was quantitatively determined using a chromameter digital color meter (T-135). Intelligent and active packaging system containing meat is placed on a flat black background with homogeneous lighting. The chromameter detector was placed on the surface of the intelligent packaging indicator. The measurement results were expressed according to the notation of the Hunter’s Lab Colorimetric System, which is presented in three values, namely L* (lightness), a* (redness), and b* (yellowness) [31]. The color of the intelligent packaging indicator was determined by calculating the Hue value by using the formula (1) below:

(1) $$\mathrm{Hue}\;\left(\mathrm{degrees}\right)=\tan^{-1}\frac{b*}{a*}$$

where Hue (360 degrees in a circle) represents the parameters for color range, a* is a red-green mixed color, and b* is a yellow-blue mixed color.

##### Antimicrobial Activity of the Active Packaging Films 

The antimicrobial activity of the active packaging films was determined via the agar diffusion method. Each active packaging film was cut into 5 mm circles in a sterile environment and then placed on NA agar media with 0.1 mL of the test microorganism culture (*Staphylococcus aureus*) containing 10^6^ CFU/mL. Petri dishes were incubated for 24 h at 37 °C. After incubation, the inhibitory zone was measured using a caliper, this measurement was carried out with three replicates [32]. 

##### Determination of pH of the Beef Samples

The pH of the beef samples was measured using a pH meter (Oakton pH 510). First, 5 g of crushed meat was introduced with 45 mL of distilled water until the mixture became homogenous. The pH meter’s electrode was then immersed in the beef suspension until the pH value on the monitor became constant. This measurement was carried out with three replicates.

##### Measurement of TVBN

The method applied in this research referred to AOAC [33]. A Conway cup with an outer diameter of 10 cm and an inner diameter of 5 cm was utilized in this study. First, 30 mL of 7% TCA solution was added to a meat sample (10 ± 0.1 g) and mixed before filtering. A total of 1 mL boric acid solution was placed in the “inner chamber” of the Conway dish. The lid of the cup was placed in such a way that it almost covered the cup. The 1 mL filtrate was placed into the outer chamber of the Conway dish. Afterward, 1 mL saturated K_2_CO_3_ solution was put into the outer chamber. The cup was closed and rotated to mix the two liquids in the outer chamber. The blank solution was prepared following the same process but with 7% TCA instead of the filtrate. The solutions were stored at 37 °C for 2 h. Then, 2 drops of methyl red and bromothymol blue (2:1) were added to the inner Conway cup and then titrated with 0.01 N HCl until a pink hue was formed. TVBN was calculated by formula (2) as follows:

(2) $$\mathrm{TVBN}\;\mathrm{content}\;\left(\frac{\mathrm{mg}}{100\;g}\right)=\frac{\left(\mathrm{Vc}-\mathrm{Vb}\right)\times 14.007\times \;\mathrm{df}\;\times 100}{W}$$

where Vc is the volume of the HCl solution used in sample titration, Vb is the volume of the HCl solution used in blank titration, N is the normality of the HCl solution, W is the sample’s weight (g), 14.007 is the molecular weight of nitrogen, and df is the dilution factor. This measurement was carried out with three replicates.

##### Measurement of Total Plate Count 

The total amount of microorganisms was determined via the total plate count (TPC) method described in Indonesian National Standard (SNI) 2332.3: 2015. First, 1 g of the sample was added to a test tube containing 9 mL of physiological solution until homogeneous (10^−1^ dilution). The dilution was continued until 10^−6^, at which point 1 mL of the diluted sample was inoculated on NA media in duplicate via the pour plate technique. After the media solidified, the Petri dishes containing the media and the sample solution were incubated upside down at 30 °C for 48 h. Afterward, TPC was calculated using the formula (3) below [34]:

(3) $$N=\frac{\sum C}{\left[\left(1\times n_{1}\right)+\left(0.1\times n_{2}\right)\right]\times \left(d\right)}$$

where N is TPC (CFU/mL), ∑C is the number of colonies counted in all Petri dishes, n_1_ is the number of colonies counted in all Petri dishes at first dilution, n_2_ is the number of colonies counted in all Petri dishes at second dilution, and d is the dilution factor corresponding to the first dilution.

#### 2.2.6. Data Analysis

ANOVA was used to analyze the parameters of the intelligent packaging indicator, antimicrobial activity of the active packaging films, and quality of the beef samples, including pH, TVBN, and TPC. Differences between treatments were determined using Duncan’s test. The correlations between the changes in the color of the intelligent packaging indicator and the effects of the active packaging on all parameters of meat spoilage were explored and presented in graphs by using the Sigma Plot 12 software. Data were analyzed using Microsoft Excel 2019, SPSS 19, and Sigma Plot 12.

## 3. Results and Discussion

### 3.1. Antimicrobial Activity of the Active Packaging Films against Staphylococcus aureus

**Figure 2:**
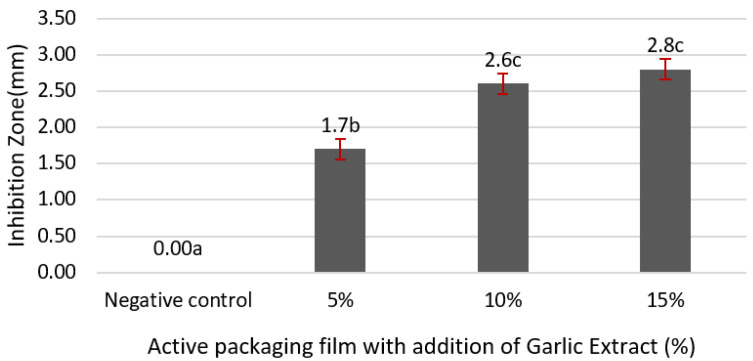
Antimicrobial activity of the active packaging films against *S. aureus*. The mean value followed by different letters showed a significant difference based on the Duncan’s test at the 5% level (*p*-value < 0.05).

The antimicrobial activity of the active packaging films is presented in Figure 2.

The antimicrobial activity of the active packaging films against *S. aureus* was assessed by measuring the diameter of the inhibition zone. As shown in Figure 2, the negative control did not generate an inhibitory zone. However, when high concentrations of the garlic extract were added to the active packaging films, the inhibitory activity against the bacteria increased, although the inhibition zone was not significantly different between 10% and 15% garlic extract. This study demonstrated that 10–15% garlic extract has antibacterial effects. According to Maroles et al. [35], differences in the diameter of inhibitory zones are influenced by the ability and rate of diffusion of antimicrobial compounds in the medium, the growth rate of microorganisms and their sensitivity to antimicrobial chemicals, and the viscosity and thickness of the medium. 

The antibacterial effects of garlic extract are due to allicin, which is generated when garlic is damaged. When the flesh of garlic is damaged during the refining process, allicin is rapidly generated because of the release of alliinase, which reacts with nonprotein amino acids, namely, alliin. Allicin is a part of the defense mechanism of garlic that exerts antimicrobial effects on both Gram-positive and Gram-negative bacteria by inhibiting RNA and lipid syntheses, which in turn inhibit the production of amino acids and proteins and the phospholipid bilayer of bacterial cell wall, thereby preventing bacterial growth and development. Allicin is highly permeable and can easily penetrate bacterial cells across the cell membrane. The thiosulfinate S(=O)S group in allicin then binds to the sulfhydryl groups of bacteria, thus inhibiting the activation mechanism of bacterial proteinases [36,37].

### 3.2. pH of the Beef Samples

The pH of the beef samples was measured to investigate the effects of the active packaging films as the meat base in the packaging system. The beef samples were stored at room temperature for 24 h. The results of pH measurements are shown in Table 1.

A statistical test of the storage time showed a significant difference to the pH value (0.000 < 0.005). However, the statistical test results of the active packaging (0.654 > 0.005) and interaction between active packaging and storage time (0.179 > 0.005), on the other hand, did not show a significant effect on the pH value (Table 1). One of the characteristics that contribute to meat quality reduction is pH. However, pH cannot be used as the single indicator of meat rot. The pH value is used to confirm the results of other meat deterioration parameters such as TPC or TVBN. According to statistics, active packaging had no significant influence on the pH of the meat and the change in pH seemed to fluctuated, but the data still indicated a rise in pH at each increase in time.

The initial pH of the meat samples, which was immediately determined after the cow was slaughtered, was normal (6.57) (Table 1). The pH fluctuated during the storage period, but the trend graph has shown a decrease in pH at 12 h then the pH increased at the 16 h to 24 h storage. After the animal dies, the blood flow that supplies oxygen to the muscles stops causing an anaerobic glycolysis process to occur. During anaerobic glycolysis, glycogen conversion occurs in the muscles to lactic acid which accumulates in the tissues, causing the pH of the meat to decrease (4 h storage), during anaerobically glycolysis, the decrease in pH continues until the glycogen is converted to lactic acid followed by the neutralization of alkaline compounds resulting from the metabolism of microorganisms, so that the pH of the meat rises again (16–24 h storage).

According to Sánchez-Macías et al. [38] and Moreno et al. [39], reported that the lower the content of glycogen in the meat is, the slower the glycolysis process will be and the higher the final pH will be. However, the decrease in pH in muscles can be influenced by internal factors, such as species, muscle type, muscle glycogen content, and livestock variability, as well as external factors, such as environmental temperature, additional treatment prior to slaughter, and pre-slaughter stress. 

After 20 h of storage, the meat’s pH value ranged from 6.75 and 6.85 and remained steady up to 24 h of storage; at this point, the meat was classified as decayed (Table 1). According to Prache et al. [40], the meat’s pH continues to decline until glycogen is depleted into lactic acid and alkaline compounds are neutralized because of microbial metabolism, resulting in an increase in pH. If the pH reaches 6.8 or higher, protein decomposition will occur, resulting in spoilage.

### 3.3. TVBN of the Meat Samples

The TVBN values of the meat samples are presented in Table 2.

A statistical test revealed a highly significant difference between the active packaging (0.004 < 0.005) and storage time (0.000 < 0.005) on the TVBN value. However, the statistical test results of the interaction between active packaging and storage time (0.986 > 0.005), on the other hand, did not show a significant effect on the TVBN value (Table 2). At 0 h, all meat samples had TVBN values ranging from 7.23 mgN/100 g to 8.35 mgN/100 g (Table 2). Therefore, they were classified as fresh meat. After 12 h of storage, the meat samples that had not been treated with the active packaging films had a TVBN value of 20.67 mg N/100g, indicating that they were rotten. By comparison, the meat samples treated with the active packaging films and added with 5%, 10%, and 15% garlic extract had TVBN values of 16.19, 17.31, and 16.61 mgN/100 g, respectively. Thus, they were categorized as semi-fresh meat (stale meat) or could still be consumed. However, the TVBN values of all meat samples taken between the 16th to 24th h of storage exceeded the threshold for food-grade beef, demonstrating that adding 5%, 10%, and 15% garlic extract to the active packaging films effectively reduced the amount of TVBN. On the other hand, meat samples that were not treated active packaging film had a significant increase in TVBN value at 12 h storage. Beef or livestock is considered fresh if the TVBN value is less than 15 mg/100 g [41] or TVBN is <10 mg N/100 g [42]. Moreover, SNI 2354.8:2009 states that the standard levels of TVBN fit for consumption is 20–30 mg N/100 g [43].

In this study, the values of TVBN increased throughout the storage period (observed every 4 h), indicating that the meat’s quality continued to deteriorate owing to the breakdown of proteins into volatile base compounds. According to Bekhit et al. [10], the increase in TVBN value is due to protein degradation by microorganisms that results in the formation of foul-smelling chemicals, such as ammonia (NH_3_), basic skatole and indole compounds, mercaptans and H_2_S (which are weak acids), and amines and cadaverin (which are strong bases). The results demonstrated that the addition of garlic extract to the active packaging films delayed the spoiling of the meat samples likely because the garlic’s active components prevented microbial development, thereby lowering the synthesis of nitrogenous base compounds in the meat caused by bacteria and autolytic enzymes during the rotting process. This conjecture was supported by Al Hakim et al. [44] and Reiter et al. [37], who reported that garlic extract has the ability to block microbe-produced enzymes involved in the breakdown of proteins into volatile base chemicals.

### 3.4. TPC of the Microbes in the Beef Samples

The TPC of bacteria in the meat samples was determined to assess the utility of the active packaging films (Table 3).

A statistical test revealed a highly significant difference between the active packaging (0.000 < 0.005) and storage time (0.000 < 0.005) on the TPC value. However, the statistical test results of the interaction between active packaging and storage time (0.09 > 0.005), on the other hand, did not show a significant effect on the TPC value (Table 3). At 0 h of the storage period, the initial TPC value (Log TPC) of all meat samples was 2.53 ± 0.64 CFU/mL (Table 3). Thus, the meat samples were classified as fresh on the basis of microbiological quality. Throughout the storage period, the TPC value increased until it reached the maximum number of meat microbes permitted by SNI 3932:2008 on carcass and beef quality, which is 1 × 10^6^ CFU/mL or equivalent to Log TPC 6 CFU/mL. At 12 h of storage, the meat samples packaged with the control film (0%) and those added with 5% garlic extract did not fulfil the microbiological requirements as they had a Log TPC value of 7.65 ± 0.39 and 6.20 ± 0.00 CFU/mL, respectively. By comparison, the meat samples treated with the active packaging films and 10% and 15% garlic extract did not fulfil the microbiological requirements after 16 h of storage as they have a Log TPC value of 7.47 ± 0.26 and 6.78 ± 0.67 CFU/mL, respectively. This result demonstrated that the active packaging films with 10% and 15% garlic extract in the meat packaging system can inhibit microbial growth and extend the shelf life of meat by up to 4 h because allicin can inhibit the growth of both Gram-positive and Gram-negative bacteria by destroying the sulfhydryl group bound to bacterial proteins. This process is important because the sulfhydryl group is required for bacterial cell division or acts as a specific stimulator for cell multiplication. Allicin damaged the RNA and DNA of bacteria and thus inhibits their growth and development in meat. Likewise, Deresse [45] reported that allicin can suppress the growth of both Gram-positive and Gram-negative bacteria by completely inhibiting the syntheses of bacterial RNA, DNA, and proteins.

The total microbial content of the meat samples continued to increase during the entire storage period (Table 3) because meat contains a high nutrient and water content, which provides an ideal environment for microorganism growth. Moreover, storage at room temperature can accelerate the growth of microorganisms. According to Soeparno [46], meat has the ideal conditions for microorganism growth because it contains a high proportion of water (68–75%), it is rich in nitrogen-containing substances of varying complexity, it contains various fermentable carbohydrates, it is rich in minerals and essential nutrients for microorganism growth, and it has a suitable pH for microorganism growth (pH 5.3–6.5).

### 3.5. Changes in the Color of the Intelligent Packaging BTB Indicator Solution as a Measure of the Freshness of the Meat Packaged with the Active Packaging Films

**Figure 3:**
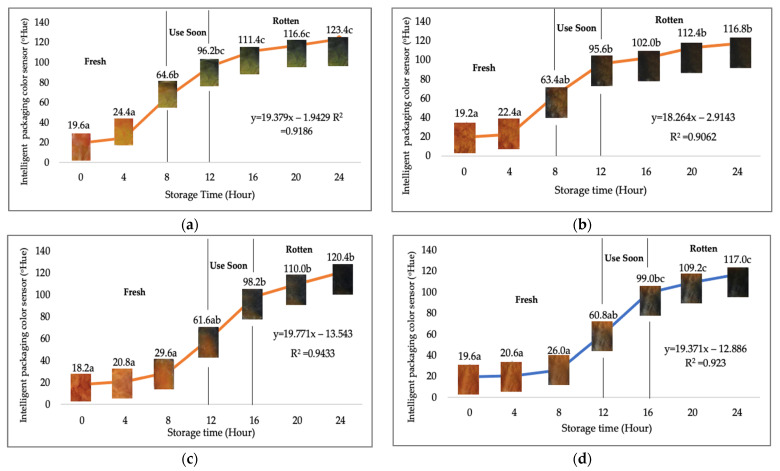
Changes in the color of the BTB solution (pH 2.75) as the intelligent packaging indicator reflecting the freshness of the meat samples packed with the active packaging films with (**a**) 0%; (**b**) 5%; (**c**) 10%; and (**d**) 15% of garlic extract. The mean value followed by different letters showed a significant difference based on the Duncan’s test at the 5% level (*p*-value < 0.05).

Using fresh beef packaged and maintained at room temperature for 24 h, Dirpan et al. [7] determined that BTB solution (pH 2.75) produces the most readily visible color changes to sensitivity tests. In this study, the BTB solution (pH 2.75), as the intelligent packaging indicator, was also utilized to evaluate changes in its color as a reflection of the freshness of the meat samples packed with the active packaging films (Figure 3).

During the entire storage period, the intelligent packaging indicator changed in three different color that corresponded to three phases of the meat samples’ level of quality (Figure 3). In phase I, its color was orange, indicating that the meat samples were still fresh. In phase II, its color was green with an orange hue, suggesting that the meat samples should be consumed immediately. In phase III, its color was dark green, denoting that the meat samples were already spoiled. The change in the indicator’s color from orange to green indicated that the quality of the meat samples had deteriorated. The changes in the indicator’s color were due to the interactions of alkaline volatile compounds produced by enzyme activity, and the metabolism of the microorganisms present in the meat samples increased with storage time. The early sign of spoilage was indicated by the release of volatile alkaline compounds as the microorganisms and the enzymes degraded the nutritional content of the meat samples. These compounds gradually accumulated in the packaging system, causing an increase in pH, which was detected by the intelligent packaging indicator and displayed as gradual color changes. The change in color of the intelligent packaging indicator (BTB, pH 2.75) from orange to green was induced by deprotonation or the release of a proton from the intelligent packaging indicator dye [47].

The meat samples packaged with the active packaging films without garlic extract (0%) and 5% garlic extract were still fresh from the start of the storage up to 8 h (Figure 3). However, they must be immediately consumed from the 8th h to the 12th h of the storage period. Thereafter (12–24 h of the storage period), they were already spoiled. This result was consistent with that of TPC tests, which showed that the TPC values were above the acceptable threshold for microbial contaminants (1 × 10^6^ or equivalent to 6 CFU/mL) in meat after 12 h. In comparison, the meat samples packaged with the active packaging films containing 10% and 15% garlic extract were still considered fresh from the start of the storage period up to the 12th h. They must be immediately consumed when they had been in storage for 12–16 h. Finally, they were considered rotten when they had been in storage for 16–24 h. This result was also consistent with that of TPC tests (Table 3), which indicated that at the 16th h, the TPC value surpassed the permissible level of microbiological contamination in beef. Statistical analysis revealed that storage duration had a very significant effect on the Hue value, the indicator of color change in the intelligent packaging. The changes in the color of the intelligent packaging indicator (BTB solution, pH 2.75) when used together with the active packaging films to reflect the freshness of meat are presented in Table 4.

### 3.6. Correlations between Changes in the Color of the Intelligent Packaging Indicator and the Effects of the Active Packaging Films on the Parameters of Meat Freshness

The correlations between changes in the color of the intelligent packaging indicator and parameters of meat quality deterioration (pH, TVBN, and TPC) were explored to ascertain the relationship between the sensitivity of the intelligent packaging indicator to meat freshness and the effectiveness of the active packaging films in slowing the process of meat spoilage.

**Figure 4:**
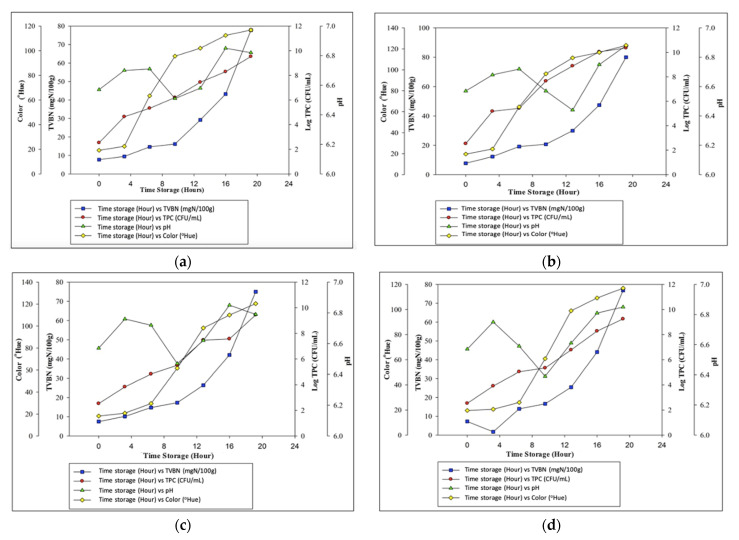
Correlations between changes in the color of the intelligent packaging indicator and the effects of the active packaging films with (**a**) 0%; (**b**) 5%; (**c**) 10%; and (**d**) 15% garlic extract on the parameters of quality deterioration of meat stored for 24 h.

Based on Figure 4, it is known that the color change of the intelligent packaging indicator which is indicated by an increase in the Hue value is in line with the increase in all values of the meat deterioration parameter. The meat samples packaged with the control film and those treated with 5% garlic extract were rotten and unfit for consumption after 12 h of storage as their Log TPC value was 7.65 ± 0.39 and 6.20 ± 0.00 CFU/mL, respectively, and their TVBN value was 20.67 ± 2.68 and 16.19 ± 0.28 mgN/100g, respectively (Figure 4). In comparison, the meat samples treated with the active packaging films and 10% and 15% garlic extract were rotten and unfit for consumption after 16 h of storage as their Log TPC value was 7.47 ± 0.26 and 6.78 ± 0.67 CFU/mL, respectively, and their TVBN value was 26.41 ± 3.31 and 25.43 ± 4.89 mgN/100 g, respectively.

Meat that was treated with active packaging film without addition (0%) and with the addition of 5% garlic extract experienced a change in indicator color from orange (fresh) with a Hue value of 19.6° and 19.20°, respectively to green (rotten) with Hue values 96.2° and 95.6°, respectively. Meanwhile, the meat that was treated with active packaging film with the addition of 10% and 15% garlic extract experienced a change in indicator color from orange (fresh) with Hue values 18.2° and 19.6°, respectively, to green (rotten) with Hue values 98.2° and 99°, respectively. Wiryawan [48] observed that when garlic extract was added to the active packaging, the values of TPC and TVBN and the pH of the meat increased more slowly, as did the color of the intelligent packaging indicator, compared with those of the meat without the active packaging.

Furthermore, the increase in the values of TPC and TVBN linearly correlates with the increase in Hue value and color changes of the intelligent packaging indicator because the accumulated volatile base compounds raise the pH value of the packaging system, causing the intelligent packaging indicator to experience a color shift. This explanation was in agreement with that of Pacquit et al. [12], who applied active packaging films to cod fish. They stated that the increase in the TPC value of cod fish has a linear correlation with changes in the color of the cellulose-acetate packaging film sensor.

On the other hand, the pH of the sample fluctuated making it difficult to determine the level of quality degradation in meat. However, the interpretation of the TPC and TVBN values, on the other hand, is clear enough to represent a decrease in meat quality which is correlated with an increase in the Hue value of changes in the intelligent packaging indicator. This good correlation demonstrates the accuracy of the film formulation in the monitoring of meat freshness, which is the aim of using intelligent packaging.

## 4. Conclusions

The paper concludes that intelligent packaging indicators using BTB (Bromothymol blue) pH 2.75 solution can be used as an indicator to identify a decline in the quality of packaged meat. The indicator’s color changes are easy to observe visually, namely the orange indicator indicating that the meat is still fresh and the dark green indicator indicating that the meat has rotted and is unfit for consumption. On the other hand, the use of active packaging can extend the shelf life of meat by 4 h longer when using high concentrations of garlic extract. This demonstrates that intelligent and active packaging, which are typically studied separately, have the potential to be combined and researched together using the same basic ingredient, namely bacterial cellulose.

## Figures and Tables

**Table 1:** pH values of packaged meat sample stored at room temperature for 24 h.

Storage Time (h)	Addition of Garlic Extract to the Active Packaging Film				Average
	0%	5%	10%	15%	
0	6.56 ± 0.08	6.56 ± 0.08	6.57 ± 0.08	6.57 ± 0.08	6.56 ± 0.00 ^a^
4	6.68 ± 0.04	6.70 ± 0.06	6.76 ± 0.10	6.75 ± 0.06	6.72 ± 0.04 ^d^
8	6.72 ± 0.05	6.71 ± 0.07	6.72 ± 0.05	6.59 ± 0.20	6.68 ± 0.06 ^d^
12	6.57 ± 0.11	6.51 ± 0.03	6.47 ± 0.04	6.39 ± 0.02	6.48 ± 0.08 ^b^
16	6.44 ± 0.23	6.58 ± 0.06	6.62 ± 0.08	6.61 ± 0.11	6.56 ± 0.08 ^c^
20	6.75 ± 0.05	6.85 ± 0.01	6.85 ± 0.02	6.81 ± 0.02	6.81 ± 0.05 ^e^
24	6.88 ± 0.11	6.82 ± 0.11	6.79 ± 0.10	6.8 5 ± 0.03	6.83 ± 0.04 ^e^

The mean value followed by different letters showed a significant difference based on the Duncan’s test at the 5% level (*p*-value < 0.05).

**Table 2:** Total volatile basic nitrogen (TVBN) of the packed meat stored at room temperature for 24 h. Average Result of Meat’s TVBN value.

Storage Time (h)	Addition of Garlic Extract to the Active Packaging Film				Average
	0%	5%	10%	15%	
	mgN/100 g				
0	8.35 ± 0.96	7.37 ± 0.56	7.51 ± 0.54	7.23 ± 1.24	7.62 ± 0.50 ^a^
4	12.27 ± 0.54	9.47 ± 2.80	10.17 ± 2.17	10.73 ± 1.17	10.66 ± 1.19 ^b^
8	19.13 ± 2.07	14.65 ± 0.72	14.79 ± 1.40	13.95 ± 0.96	15.63 ± 2.36 ^c^
12	20.67 ± 2.68	16.19 ± 0.28	17.31 ± 1.73	16.61 ± 1.21	17.70 ± 2.04 ^c^
16	29.91 ± 3.78	29.21 ± 5.57	26.41 ± 3.31	25.43 ± 4.89	27.74 ± 2.16 ^d^
20	47.41 ± 3.17	43.21 ± 1.19	42.09 ± 1.19	44.05 ± 0.79	44.19 ± 2.29 ^e^
24	80.03 ± 8.65	77.79 ± 3.11	74.99 ± 5.63	76.81 ± 8.26	77.42 ± 2.12 ^f^
**Average**	31.12 ± 25.13 ^b^	28.27 ± 25.16 ^a^	27.61 ± 23.93 ^a^	27.83 ± 24.84 ^a^	

The mean value followed by different letters showed a significant difference based on the Duncan’s test at the 5% level (*p*-value < 0.05).

**Table 3:** Total plate count (TPC) of packed meat stored at room temperature for 24 h.

Storage Time (h)	Addition of Garlic Extract to the Active Packaging Film				**Average**
	0%	5%	10%	15%	
	log CFU/mL				
0	2.53 ± 0.64	2.53 ± 0.64	2.53 ± 0.64	2.53 ± 0.64	2.53 ± 0.00 ^a^
4	5.18 ± 0.20	4.64 ± 0.16	3.83 ± 0.30	3.91 ± 0.10	4.39 ± 0.64 ^b^
8	5.43 ± 0.21	5.33 ± 0.07	4.83 ± 0.40	5.04 ± 0.06	5.16 ± 0.27 ^c^
12	7.65 ± 0.39	6.20 ± 0.00	5.51 ± 0.10	5.34 ± 0.08	6.18 ± 1.05 ^d^
16	8.89 ± 0.67	7.44 ± 0.03	7.47 ± 0.26	6.78 ± 0.67	7.64 ± 0.89 ^e^
20	10.04 ± 0.58	8.30 ± 1.35	7.57 ± 0.60	8.28 ± 0.06	8.55 ± 1.08 ^f^
24	10.36 ± 0.15	9.53 ± 0.39	9.44 ± 0.68	9.25 ± 0.03	9.65 ± 0.49 ^g^
**Average**	7.15 ± 2.89 ^c^	6.28 ± 2.37 ^b^	5.88 ± 2.41^a^	5.88 ± 2.39 ^a^	

The mean value followed by different letters showed a significant difference based on the Duncan’s test at the 5% level (*p*-value < 0.05).

**Table 4:** Changes in the color of the intelligent packaging indicator (BTB solution, pH 2.75) when used together with the active packaging films to reflect the freshness of meat.

Storage Time(h)	Active Packaging Films Added with Garlic Extract			
	0%	5%	10%	15%
0	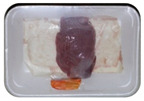	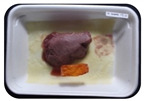	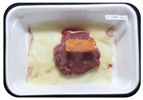	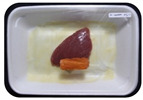
4	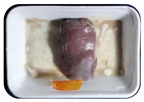	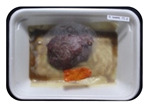	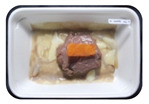	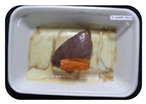
8	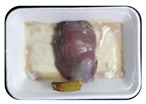	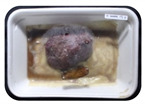	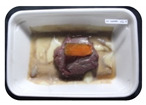	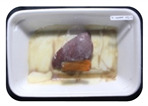
12	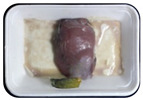	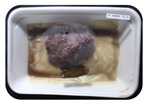	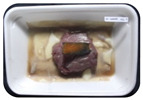	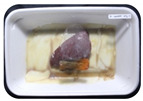
16	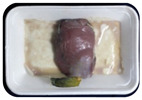	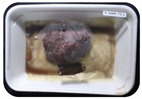	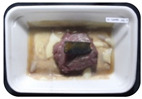	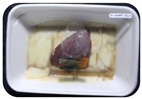
20	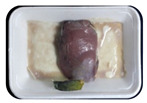	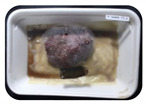	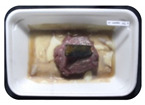	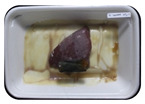
24	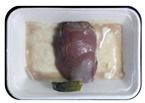	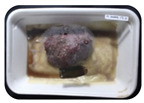	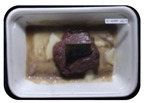	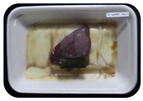

## Data Availability

Available data are presented in the manuscript.
